# Evolution of an endofungal Lifestyle: Deductions from the *Burkholderia rhizoxinica *Genome

**DOI:** 10.1186/1471-2164-12-210

**Published:** 2011-05-04

**Authors:** Gerald Lackner, Nadine Moebius, Laila P Partida-Martinez, Sebastian Boland, Christian Hertweck

**Affiliations:** 1 Leibniz Institute for Natural Product Research and Infection Biology (HKI), Department of Biomolecular Chemistry, Beutenbergstr. 11a, 07745 Jena, Germany; 2 Departamento de Ingeniería Genética, CINVESTAV-Irapuato, Km. 9.6 Libramiento Norte, CP 36821 Irapuato, Guanajuato, México; 3 Max Planck Institute of Molecular Cell Biology and Genetics, Pfotenhauerstr. 108, 01307 Dresden, Germany; 4 Friedrich Schiller University, 07743 Jena, Germany

## Abstract

**Background:**

*Burkholderia rhizoxinica *is an intracellular symbiont of the phytopathogenic zygomycete *Rhizopus microsporus*, the causative agent of rice seedling blight. The endosymbiont produces the antimitotic macrolide rhizoxin for its host. It is vertically transmitted within vegetative spores and is essential for spore formation of the fungus. To shed light on the evolution and genetic potential of this model organism, we analysed the whole genome of *B. rhizoxinica *HKI 0454 - a type strain of endofungal *Burkholderia *species.

**Results:**

The genome consists of a structurally conserved chromosome and two plasmids. Compared to free-living *Burkholderia *species, the genome is smaller in size and harbors less transcriptional regulator genes. Instead, we observed accumulation of transposons over the genome. Prediction of primary metabolic pathways and transporters suggests that endosymbionts consume host metabolites like citrate, but might deliver some amino acids and cofactors to the host. The rhizoxin biosynthesis gene cluster shows evolutionary traces of horizontal gene transfer. Furthermore, we analysed gene clusters coding for nonribosomal peptide synthetases (NRPS). Notably, *B. rhizoxinica *lacks common genes which are dedicated to quorum sensing systems, but is equipped with a large number of virulence-related factors and putative type III effectors.

**Conclusions:**

*B. rhizoxinica *is the first endofungal bacterium, whose genome has been sequenced. Here, we present models of evolution, metabolism and tools for host-symbiont interaction of the endofungal bacterium deduced from whole genome analyses. Genome size and structure suggest that *B. rhizoxinica *is in an early phase of adaptation to the intracellular lifestyle (genome in transition). By analysis of tranporters and metabolic pathways we predict how metabolites might be exchanged between the symbiont and its host. Gene clusters for biosynthesis of secondary metabolites represent novel targets for genomic mining of cryptic natural products. *In silico *analyses of virulence-associated genes, secreted proteins and effectors might inspire future studies on molecular mechanisms underlying bacterial-fungal interaction.

## Background

The genus *Burkholderia *comprises a group of highly versatile bacteria [1] that occupy remarkably diverse ecological niches in both terrestrial and aquatic environments [2]. Their multifarious lifestyle is illustrated by various infamous pathogens, free-living non-pathogens and *Burkholderia *spp. that form mutualistic symbioses, e.g. with vascular plants [3,4] and basidiomycetes [5]. A particularly intriguing tripartite microbial interaction involving a *Burkholderia *sp. has been unveiled in the context of rice seedling blight, a severe plant disease affecting rice plants in Asia. The causative agent is the zygomycete *Rhizopus microsporus*, which uses rhizoxin, a potent antimitotic agent, as a virulence factor [6,7]. In the course of studying rhizoxin biosynthesis, we have found that this virulence factor is in fact not produced by the fungus, but by bacteria residing within the fungal cytosol [8,9]. These endofungal bacteria represent a new *Burkholderia *species, *Burkholderia rhizoxinica *[10]. A related endofungal bacterium, *Burkholderia endofungorum*, was identified as the true producer of the toxic cyclopeptide rhizonin [10,11]. These are the first observed cases where designated mycotoxins are not produced by the fungus itself, but by endosymbiotic bacteria [8,12,13]. In total we have identified eight related *Burkholderia-Rhizopus *associations from five different continents [14], which have likely undergone a parasitism-mutualism shift [15]. While there have been a growing number of reports on endofungal bacteria [16-19], the delicate and slowly growing *Rhizopus *endosymbionts organisms are the first ones that could be grown in pure culture [8,10]. Large-scale fermentation of isolated *B. rhizoxinica *could drastically improve work-up procedures and yield of even more potent antitumoral rhizoxin derivatives [20]. From an ecological point of view, the fungus and the bacteria form an intimate association where the symbiont delivers a chemical weapon to its host and in turn the symbiont profits from nutrients and spreading within fungal spores. A highly remarkable characteristic of the *Burkholderia-Rhizopus *symbiosis is that the bacterial symbiont tightly controls sporulation of the fungal host [21]. Strains that have been cured by antibiotic treatment are incapable of asexual reproduction and thus fully depend on the presence of the endobacteria. In this way, the persistence and distribution of this unique toxinogenic bacterial-fungal association is warranted. Recently, we have sequenced the whole genome of *Burkholderia rhizoxinica *HKI-0454, as a reference strain for *Burkholderia-Rhizopus *associations [22]. Here, we provide insights into the genetic tool box of an endofungal symbiont by extensive analysis of the full genome sequence.

## Results and Discussion

### Genome Properties and Comparative Genomics

**Figure 1:**
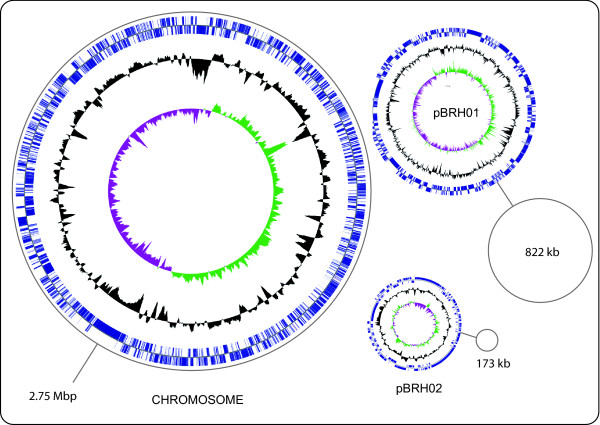
**Schematic circular diagrams of the *B. rhizoxinica *genome**. Blue: Open reading frames. Black: G+C content. Green: positive GC-skew. Violet: negative GC-skew. Simple circular representations indicate the correct size scale for megaplasmid pBRH01 and plasmid pBRH02. The chromosome exhibits a smooth GC-skew pattern, whose origin is located some 50 kb upstream of the *dna*A gene. The observed shift is presumably due to a insertion of a prophage next to the origin of replication.

The genome of *B. rhizoxinica *has a total size of 3,750,139 bp and a G+C content of 60.7%. It consists of three replicons (Figure 1) including the chromosome (2.75 Mbp), and the plasmids pBRH01 (822,304 bp) and pBRH02 (172,525 bp) [23]. Compared to other representatives of the genus *Burkholderia*, the genome is remarkably small (Table 1). Indeed, it is the smallest *Burkholderia *genome published so far, being about 50% smaller than genomes of free-living relatives. However, compared to some obligate symbionts of insects [24-27], *B. rhizoxinica *has a 3-4 times larger genome (Table 1). The whole genome consists of 3878 ORFs, 2437 (62.2%) of which were assigned a biological function. Among the 1441 ORFs without known function, 897 show similarity to other database entries, while 544 remain without function and similarity.

**Table 1:** Comparison of selected sequenced *Burkholderia *and *Wolbachia *species.

Organism (strain)	*B. rhizoxinica* HKI 0454	*B. mallei* ATCC 23344	*B. thailandensis *E264	*B. pseudomallei* K96243	*B. cenocepacia* *J2315*	*Wolbachia *sp (*w*Mel*)	***Wolbachia *sp**. TSR *(w*BM**)
Genome size (bp)	3,750,139	5,835,527	6,723,972	7,247,547	8,055,782	1,267,782	1,080,084

G+C content (%)	60.7	68.5	67.6	68.1	66.9	35.2	34.2

ORFs (total)	3827	5025	5634	5728	7269	1195	805

Transposases and derivatives	231 (6.0%)	255 (5.0%)	155 (2.8%)	85 (1.5%)	171 (2.5%)	29 (2.4%)	4 (0.5%)

Transcriptional regulators	193 (5.0%)	405 (8.0%)	517 (9.2%)	523 (9.1%)	802 (11.0%)	11 (0.9%)	8 (1.0%)

*wMel = *Wolbachia *endosymbiont of *Drosophila melanogaster*

**wBM = *Wolbachia *endosymbiont of *Brugia malayi*

**Figure 2:**
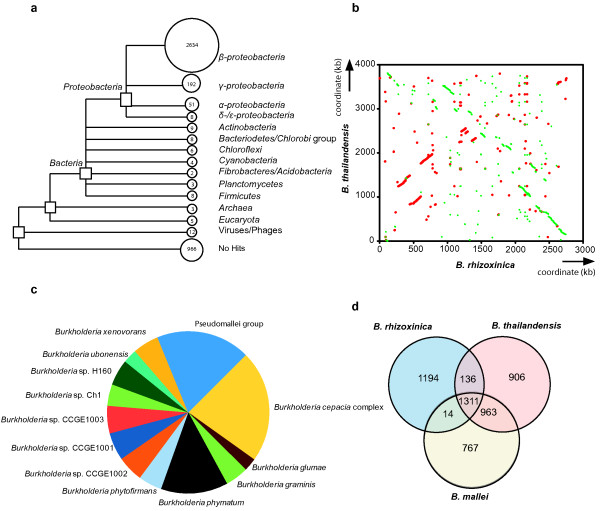
**Comparative Genomics**. a: Phylogenetic distribution of best BLASTP hits of all *B. rhizoxinica *ORFs. Organisms delivering best hits were assigned to taxonomic groups according to the NCBI taxonomy. For phylogenetic assignment and tree construction the program MEGAN 4 was used with a BLASTP cut-off score of 60. b: Synteny plot of the *Burkholderia rhizoxinica *chromosome I against the *B. thailandensis *E264 chromosome I based on a six-frame translation of the genome. Forward matches are indicated in red, reverse matches are indicated in green. c: Pie chart of the phylogenetic distribution of best BLASTP hits of all *B. rhizoxinica *ORFs within the genus *Burkholderia*. d: Venn diagram showing the number of ortholog clusters (bidirectional best BLAST hit, P < 10^-5^) among the predicted proteoms of *B. rhizoxinica *(symbiont), *B. thailandensis *E264 (soil bacterium) and *B. mallei *ATCC 23344 (pathogen).

Whole genome BLASTP analysis confirmed that the phylogenetic origin of >67% of deduced ORFs lies within the β*-*proteobacteria (Figure 2a). The distribution of best hits within the genus *Burkholderia *is shown in Figure 2c. Hits to other phyla might suggest some horizontal gene transfer. Five deduced proteins have best hits to eukaryotic genes, however, these do not deliver evidence for horizontal transfer of host genes to the symbiont genome.

Synteny plots of the chromosome indicate that the endobacteria possess a typical member of the chromosome I family of *Burkholderia *(Figure 2b). The plasmids pBRH01 and pBRH02 appear to represent strain-specific elements. Because of its relatively small size and its lack of structural RNA genes, we regard the megaplasmid as a plasmid rather than a secondary chromosome, which are regularly found in *Burkholderia *genomes [28]. Much like other secondary chromosomes and plasmids of *Burkholderia *species, they presumably depend on a ParA/B partitioning system [29]. ParB protein encoded on pBRH01 aligns well with homologs found on other *Burkholderia *replicons. However, a phylogenetic analysis (Additional file 1, Figure S1) did not reveal further details about the origin of the megaplasmid, suggesting that it has split off early in the evolution of *Burkholderia *plasmids and chromosomes. ParB of plasmid pBRH02 is significantly more distantly related to other known partitioning proteins with its closest homolog found in the γ-proteobacterium *Enhydrobacter aerosaccus *(Additional file 1, Figure S1).

In order to obtain a subset of gene families characteristic for endofungal symbionts we conducted comparative analyses by computation of similarity clusters with related *Burkholderia *species. As an example we chose the apathogenic, free-living soil bacterium *B. thailandensis *E264 and the obligate animal pathogen *B. mallei *for comparative studies (Figure 2d). While 1311 ortholog clusters are shared by all three organisms, 1194 ORFs are exclusively present in *B. rhizoxinica *encompassing 30.8% of its genes. *B. rhizoxinica *specific genes comprise 939 (78,6%) hypothetical proteins. We assume that some strain-specific genetic material of unknown function is associated with the development of endofungal lifestyle. Among the 255 genes with a proposed function, a large proportion appears to be mobile genetic elements such as 55 transposase genes (21, 6%), 77 phage component genes (30, 2%) and 16 genes coding for plasmid stability systems (6,3%).

### Mobile Genetic Elements and Pseudogenes

The *B. rhizoxinica *genome is disrupted by a considerable amount of potentially mobile genetic elements with 231 ORFs (6.0%) encoding proteins with similarity to transposases or inactivated derivatives. Although it is unknown how many of these form parts of active insertion elements, this number is high compared to other sequenced *Burkholderia *species (Table 1). This finding is in agreement with the hypothesis that intracellular bacteria have a tendency to accumulate transposons in early stages of the symbiosis [30]. Besides the shear number of transposons, there are numerous genes for plasmid stability proteins, reverse transcriptases, and phage proteins. The genetic evidence supporting recent exposure to foreign DNA contrasts obligate endosymbionts like *Buchnera *[25,27,30] that have been living genetically isolated for a long evolutionary period. *B. rhizoxinica *contains several predicted toxin-antitoxin (TA) systems including RelE, MazF and PIN domain proteins encoding genes. These factors mediate stability of plasmids and other mobile elements by imposing an addiction-like mechanism to the host and are wide-spread in prokaryotic genomes [31]. However, besides their rather selfish role in genetic addiction these factors have been proposed to mediate growth retardation under stress conditions [32]. Thus, the abundance of TA systems might be important for endofungal growth control in certain stages of host colonization.

The high number of insertion elements combined with reduced genome size delivers evidence that the *B. rhizoxinica *genome is in a process of genome erosion. In some endosymbionts like *Sodalis glossninidius*, a maternally transmitted symbiont of tsetse flies, genome erosion is connected to a high number of pseudogenes [33]. Indeed, especially genes coding for mobile genetic elements appear to be truncated or disrupted in the *B. rhizoxinica *genome as well. However, an exact estimation of the number of pseudogenes is difficult, since well studied reference organisms with a comparable gene set are not available.

### Primary Metabolic Capabilities and Uptake of Nutrients

Two of the key questions concerning the endofungal lifestyle of *B. rhizoxinica *are, "How do endobacteria exchange metabolites with their fungal host and are they producers or consumers of certain nutrients and cofactors?". To conceive a model for metabolic processes that may govern the intracellular lifestyle of *B. rhizoxinica*, we deduced metabolic pathways for primary metabolism, cofactor biosynthesis and membrane transport. From previous studies, it was known that *B. rhizoxinica *does not consume glucose, but instead prefers glycerol as a carbon source in pure culture [10]. In accordance to this finding, we found three genes encoding glycerol kinases and a putative glycerol uptake facilitator. On the contrary, no genes for glucose transporters could be found in the genome [23]. Since we could deduce all enzymes required for a functional Entner-Doudoroff pathway, we suspect that this pathway is used for gluconeogenesis. Furthermore, genes dedicated to ethanol assimilation and import of organic acids such as citrate and C4-dicarboxylates (malate, fumarate, succinate) are present in the genome. This finding suggests that *B. rhizoxinica *can take up citrate, malate, glycerol or ethanol during its intracellular life and it has been reported that *Rhizopus *produces ethanol and glycerol when grown on various sugars or wood hydrolysates [34]. Regarding intracellular energy metabolism, the presence of a gene coding for a putative ATP/ADP antiporter (RBRH_01687) on the megaplasmid is intriguing and it might enable endobacteria to directly withdraw energy equivalents from the cytosol of their host. While missing in other sequenced *Burkholderia *strains, such specialized forms of antiporters are found in obligate intracellular pathogens such as *Rickettsia *[35] or the plant pathogen *Xylella fastidiosa *[36]. The presence of the ATP/ADP antiporter demonstrates the thin line between parasitic and mutualistic lifestyle.

For amino acid metabolism, *B. rhizoxinica *harbors genes responsible for importing branched-chain and aromatic amino acids, histidine, acidic amino acids and glycine. Furthermore, the genome encodes biosynthetic routes for all proteinogenic amino acids and efflux systems for basic amino acids and cystein. We suspect that amino acid transporters were adapted to the metabolic situation during symbiosis. In addition to amino acid transporters, the endosymbiont genome contains genes for dipeptide and oligopeptide import systems. *B. rhizoxinica *harbors genes dedicated to the biosynthesis of the essential cofactors like pyridoxin, heme, flavin, biotin and thiamine. Combined with the previously mentioned amino acid pathways, this finding reflects the observed ability of isolated strains to grow in relatively nutrient poor mineral salt media. Transport processes for cofactors, however, could not be inferred by genomic data, except for a putative cobalamin transport system. Concerning trace elements, *B. rhizoxinica *harbors uptake systems for iron-siderophores, iron, magnesium/cobalt, manganese, zinc, potassium and a sodium/calcium antiporter.

### Secondary Metabolic Potential

*B. rhizoxinica *is known for its capacity to produce the antimitotic polyketide rhizoxin, the virulence factor of *R. microsporus *[8,20]. The genomic locus for rhizoxin biosynthesis (*rhi*) that encodes a hybrid system of nonribosomal peptide synthetase and polyketide synthase (NRPS/PKS), was recently discovered [9]. Functional analyses unveiled several unprecedented features of this thiotemplate system [37-39]. Full genome sequencing has now revealed that the *rhi *locus is encoded on the large chromosome and neither of the megaplasmids, however, since the *rhi *gene cluster is flanked by transposase genes, it may represent a potentially mobile region of the genome.[23]. Additionally, a highly homologous gene cluster has been discovered in *Pseudomonas fluorescens *Pf-5 and shown to be responsible for rhizoxin production [40,41]. Analysis of compositional nucleotide bias and its location near the chromosomal terminus of replication further supports the hypothesis that the gene cluster has been horizontally acquired by *B. rhizoxinica*. However, the overall G+C content clearly disagrees with a direct transfer between ancestors of the two species. While the G+C content of the *B. rhizoxinica *gene cluster is lower than the genome average (58.3% versus 60.2%), the G+C content of the *P. fluorescens *cluster is higher than the genome average (65.5% versus 63.3%). From a phylogenetic point of view it is intriguing that we encountered a stretch of 160 bp (*rhiF*') on the megaplasmid exhibiting high sequence similarity with the end of the rhizoxin biosynthesis gene cluster (*rhiF)*. Hence, we suspect that the rhizoxin gene cluster might have been transferred from the megaplasmid to the chromosome, where it effectively represents an insertion of a genomic island. The neighboring gene of *rhiF' *on the megaplasmid codes for a putative drug exporter of the major facilitator superfamily (MFS) [42], which has a homolog in *P. fluorescens *Pf-5. Therefore, it is possible that this gene is involved in rhizoxin export and was previously part of the *B. rhizoxinica rhi *gene cluster. However, *B. rhizoxinica *harbors a variety of efflux pumps including macrolide-specific ABC-transporters (see below), which could accomplish toxin delivery to the host fungus. Furthermore, NRPS/PKS systems require a phosphopantetheinyl transferase (PPtase) responsible for activation of the acyl and peptidyl carrier protein domains. Whole genome data enabled us to identify a single open reading frame encoding a PPtase [43] on the megaplasmid (*brp*, RBRH_02776) that may function in this capacity. Apart from the conserved holo-ACP synthase (ACPS) gene, which is typically essential for the activation of fatty acid synthases, this is the only gene with significant similarity to known PPtase genes.

**Figure 3:**
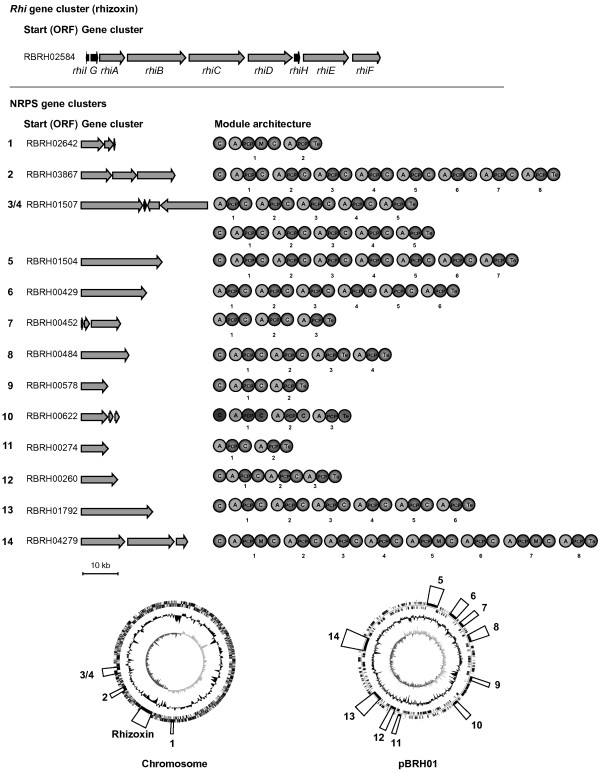
**Representation of each PKS (polyketide synthase) and NRPS (nonribosomal peptide synthetase) gene cluster discovered on the genome of *B. rhizoxinica***. Arrows represent ORFs and indicate direction of transcription, circles represent domains of deduced NRPS enzymes. A = adenylation domain responsible for recognition and activation of the amino acids. C = condensation domain (catalyzes the peptide bond formation). T = peptidyl carrier protein (carries the growing peptide chain bond as a thioester.

In addition to the rhizoxin biosynthesis gene cluster, *B. rhizoxinica *harbors 14 non-ribosomal peptide synthetase (NRPS) gene clusters (Figure 3). NRPSs are giant multimodular enzymes [23]. Each module typically catalyzes incorporation of one amino acid into the peptide product [44]. The gene clusters and the module architecture of their corresponding NRPS systems are summarized in Figure 3. There are two gene clusters coding for octamodular NRPSs. One of the deduced assembly lines is equipped with three additional methylation domains that catalyze *N*-methylation of the incorporated amino acid. Another NRPS is encoded by the longest ORF in the genome, which has a total length of 23.3 kb. Notably, the total DNA of all PKS and NRPS loci sums up to 322 kb, covering 9% of the whole genomic sequence. In addition, the megaplasmid bears a gene related to lantibiotic biosynthesis (RBRH_00226), whose closest homolog is found in cyanobacteria [45]. However, its function is unclear, since it is not flanked by genes for secretion or leader peptide processing. Taken together, this considerable biosynthetic potential is surprising, since no peptides corresponding to the gene clusters could be isolated from *B. rhizoxinica *up to now. We hypothesize that some NRPS products may function as siderophores or antibiotics. It is well conceivable that peptides serve as signal molecules, effectors in bacterial-fungal interaction or support virulence of the phytopathogenic host fungus.

### Lipopolysaccharides and Exopolysaccharides

Carbohydrate-derived surface determinants of Gram-negative bacteria, such as lipopolysaccharides (LPSs) and exopolysaccharides have a major impact in recognition processes [46] and could also play a role in the *Burkholderia-Rhizopus *symbiosis. Recently, we have identified a gene cluster that is involved in lipopolysaccharide (LPS) biosynthesis in *B. rhizoxinica *and solved the chemical structure of the LPS [47]. The homopolymeric O-antigen consists of 2-substituted D-galactofuranose, [→2)-β-D-Gal*f*-(1→]_n _and has been shown to be important for stability of the symbiosis. Deletion of the O-antigen ligase gene *waaL *resulted in drastically decreased ability of endosymbionts to form stable symbiosis with host fungi. Besides possessing genes responsible for the biosynthesis of homopolymeric O-antigen LPS, which is dependent on an ABC- transporter [48], genome sequencing revealed genes that could enable *B. rhizoxinica *to produce exopolysaccharide (EPS) or type I capsular polysaccharides [49]. Such a system requires the antigen flippase and polymerase genes *wzx *and *wzy *as well as genes for the trans-envelope transport complex Wza, Wzb, Wzc, and a dedicated glycosyl transferase. The homologs of Wzx (RBRH_01445, RBRH_03897), Wzy (RBRH_00521) and Wzb (RBRH_03824) are encoded on different loci on the chromosome. On the megaplasmid we identified a locus for capsular or exopolysaccharide biosynthesis including two glycosyltransferase genes dedicated to undecaprenyl phosphate loading, the transport complex genes *wza *(RBRH_00520), *wzc *(RBRH_00521) and a putative mannosyl transferase gene (RBRH_00522). Surface polysaccharides represent important symbiosis factors in some rhizobia [50,51] and we therefore suspect that they also mediate or support the *Burkholderia-Rhizopus *symbiosis.

### Antibiotic Resistance and Efflux Pumps

*Burkholderia rhizoxinica *is naturally resistant against various β-lactams including ampicillin, penicillin G and meticillin, but not against imipenem [10]. This result gives hints about the substrate specificity of a β-lactamase (RBRH_02672) encoded in the genome that is related to other *Burkholderia *β-lactamases [52]. Furthermore, *B. rhizoxinica *has the genetic repertoire for a broad variety of efflux pumps, including four members of the RND superfamily, six members of the ATP binding cassette (ABC) superfamily and five representatives of the major facilitator superfamily (MFS) (Additional file 2, Table S1). These systems transport low molecular weight compounds out of the cytosol of gram-negative bacteria, thus conferring resistance to antibacterial substances [42]. It is possible that efflux pumps are essential for an endofungal lifestyle, since they might expel fungal defense molecules. Another hypothetical function of efflux pumps of the RND superfamily is the export of virulence factors (type I secretion), a process that might be relevant for bacterial-fungal interaction.

### Protein Secretion Systems and Effector Proteins

One important mechanism of host control is secretion of effector proteins into host cells. Thus, we examined the genome for genes coding for further putative protein secretion systems, which are likely to be involved in host infection and control. *B. rhizoxinica *contains both the Sec and Tat pathways for transport of proteins into the periplasm. One route of export is the general secretion pathway or type II secretion [53] pathway that is present in *B. rhizoxinica *(RBRH_00771 - RBRH_00782). A type III secretion system (T3SS), whose coding gene cluster is located on the megaplasmid, has already been shown to play a crucial role for the establishment of the symbiosis [54]. T3SSs form huge membrane complexes that typically secrete effector proteins directly into the cytosol of eukaryotic host cells. A putative type IV secretion system (T4SS) is encoded on the 172 kb plasmid pBRH02 [23]. T4SS are related to conjugation machines (*tra *genes) and deliver DNA and/or proteins into recipient cells [55]. Notably, the *B. rhizoxinica *T4SS is related to a plasmid encoded T4SS from *B. cenocepacia *J2314. This system was shown to be involved in a plant pathogenicity phenotype of *B. cenocepacia *[56].

In order to understand the molecular mechanisms underlying the bacterial-fungal interaction particular proteins secreted by the various secretion systems need to be identified. Generally, T2SS are dedicated to the secretion of toxins and lytic proteins [53] into the extracellular milieu. To address this question we performed a prediction analysis of proteins bearing a signal peptide for Sec-dependent export to the periplasm (Additional file 3, Table S2). Though the presence of a signal peptide is a general feature of both periplasmatic and secreted proteins, it can give hints on putative type II secreted proteins. Intriguingly, we found chitinase (RBRH_02666), chitosanase (RBRH_02856) and a putative chitin-binding protein (RBRH_01547) in the pool of Sec-exported proteins. Given the fact that chitin is a constituent of the fungal cell wall [57,58], it is plausible that chitinase is involved in the penetration of fungal hyphae during infection. A chitinase (ChiA) has already been shown to be secreted via a type II secretion system in *E. coli *K-12 [59]. Chitinolytic enzymes could furthermore influence cell wall remodeling during growth or sporulation of *R. microsporus *as reported for *Aspergillus *species [57]. The chitin-binding protein might facilitate symbiont attachment to fungal cell walls or assist in directing chitinolytic enzymes to their substrate. In addition, the signal peptide prediction revealed putative virulence factors including peptidyl-prolyl *cis-trans *isomerases (RBRH_01604, RBRH_03962). These enzymes have been shown to enhance virulence of plant and human pathogens [60,61]. Also, a tetratricopetide protein (RBRH_03961) and an ankyrin repeat protein (RBRH_01579) bear signal peptides. Both protein families are involved in protein-protein interaction and represent good candidates for host interaction factors [62]. Notably, ankyrin repeat proteins are abundant in *Wolbachia *symbionts of insects [63].

Furthermore, we performed a prediction of type III secreted proteins encoded in the genome using the EFFECTIVET3[64] software package (Additional file 4, Table S3). Though these results have to be interpreted with cation, the prediction suggests that key enzymes of essential metabolic pathways are secreted. Among them are enzymes catalyzing crucial steps in purine and pyrimidine biosynthesis (e.g. aspartate carbamoyl transferase), as well as glucose-6-phosphate dehydrogenase, the gatekeeper to pentose phosphate pathway, which delivers ribose building blocks and NADPH for nucleotide biosynthesis. Another predicted effector protein, ribonucleotide reductase, is a key enzyme in DNA synthesis, while serine hydroxymethyltransferase produces 5,10-methylene tetrahydrofolate, which is an important source of C1 units for purine and thymidylate synthesis. Furthermore, the first enzyme for branched-chain amino acid biosynthesis (acetolactate synthase) and enzymes for aromatic amino acid (chorismate synthase), as well as arginine biosynthesis, are predicted to be released into the host's cytosol. In addition, the predicted type III secretome comprises enzymes for heme biosynthesis. Last but not least, some predicted type III effectors represent constituents of the mevalonate-independent isoprenoid pathway including the gate-keeping 1-deoxy-D-xylulose 5-phosphate synthase and a putative squalene-hopene cyclase. These findings suggests that terpenoids (e.g. ergosterol) might play a role during bacterial-fungal interaction.

The prediction of factors that are substrates of the plasmid encoded T4SS is not feasible with current bioinformatic methods. However, the plasmid carries a gene cluster encoding three large hypothetical proteins without assigned function. One of them (RBRH_03371) has a single phospholipase D domain. Phospholipase D is important in eukaryotic signaling pathways and known to be secreted by pathogens like *Corynebacterium pseudotuberculosis *[65]. However, we cannot exclude that the T4SS simply contributes to mobilization of the plasmid pBR02.

### Pilus Formation and Adhesion Proteins, and Insecticidal Toxin Complexes

*B. rhizoxinica *harbors several gene clusters related to pilus formation and adhesion. We identified several genes coding for biogenesis proteins of type IV pili including pilin precursor (PilA, RBRH_01328), NTP-binding protein (PilB, RBRH_03238), signal peptidase (PilD, RBRH_03240), secretin (PilQ, RBRH_02326) and ATPase (PilM, RBRH_02329) scattered over the chromosome. Type IV pilus-like structures might play a role in adhesion to host surfaces. However, the pilus lacks motor protein PilT and other accessory proteins. This explains why *B. rhizoxinica *cannot use pili for twitching motility. In addition, there is a locus on the chromosome encoding a fimbrial usher (RBRH_01934) protein [66]. These outer membrane proteins mediate export of folded proteins across the outer membrane, often involved in assembly of surface structures like fimbriae.

Furthermore, we identified adhesion dedicated genes coding for proteins related to hemolysins or filamentous hemagglutinin-like proteins (RBRH_00100, RBRH_04156 and RBRH_03315). They are clustered together with their corresponding secretion proteins forming a two-partner or type Vb secretion system. The giant (ca. 3 kb) hemolysin gene products are remotely related to a HecA-homolog from *Xylella fastidiosa*, which has been shown to be involved in plant pathogenicity [67]. Small parts of these genes are duplicated in the genome of *B. rhizoxinica*, possibly representing nonfunctional repetitive elements. Intriguingly, one hemolysin gene cluster is located next to the rhizoxin biosynthesis locus on the chromosome. Additionally, we identified two putative surface-associated proteins (RBRH_03838, RBRH_03272), which contain YadA domains and thus represent potential autotransporters or type Va secretion systems. [68]. It is therefore conceivable that endobacteria require a repertoire of surface proteins for their attachment to fungal surfaces during infection and transmission processes.

Another rather unexpected finding, again in the neighborhood of the rhizoxin locus (RBRH_02570 - RBRH_2567), is a gene cluster coding for homologs of an insecticidal toxin complex (tc) from *Photorhabdus luminescens *[69]. Though the presence of another tc cluster on the megaplasmid (RBRH_00589 - RBRH_00598) might be an indication for the relevance of these proteins, their role in endofungal bacteria is unclear.

### Transcriptional Regulators and a Lack of Quorum Sensing Systems

The *B. rhizoxinica *genome contains 193 transcriptional regulator genes (5% of all genes). This number is low when compared to genomes of free-living bacteria, which contain about 10% regulator genes (Table 1). This finding likely reflects a reduced need of endobacteria to adjust to varying environmental stresses and conditions. Compared to highly adapted endosymbionts, however, the loss of regulators is moderate. The *Wolbachia *endosymbiont of *B. malay*i *(w*BM), for instance retained only eight (1%) genes coding for transcriptional regulators, thus exhibiting a tremendously stronger loss of regulatory elements, which further supports the 'genome in transition' hypothesis for *B. rhizoxinica*.

Toxin production and host manipulation by bacteria often depends on quorum sensing (QS), [70], a process where bacteria sense their own density or secretion efficiency by small molecules like *N*-acyl-L-homoserine lactones (AHLs). Though these processes have been suggested to trigger toxin production and host interaction of *B. rhizoxinica *[71], its genome apparently lacks common genes responsible for the synthesis of quorum sensing autoinducers. Homologs of the AHL synthase gene, *luxI*, are regularly found in related *Burkholderia *species [72], yet they are missing in *B. rhizoxinica*. Furthermore, no regulators of the *luxR *family possessing the autoinducer binding domain (pfam03472) could be identified in the genome. Only three genes encoding regulatory proteins with a C-terminal LuxR DNA-binding domain are present. However, a more extensive search for other known QS system components did not reveal any QS candidate genes, either. The endosymbionts lack homologs of *luxM *and *luxS*, which are responsible for the biosynthesis of autoinducer-1 (AI-1) and AI-2 in *Vibrio harveyi *[70], respectively, as well as the Phc cell density sensing system found in *Ralstonia *[73] and *Cupriavidus *[74]. Neither genes for biosynthesis of quinoline signal molecules known from *B. thailandensis *nor a close homolog of BCAM0581 responsible for cis-2-dodecenoic acid formation in *B. cenocepacia *[75] could be identified. The role of QS in endosymbiosis is not fully understood. Primary and many secondary endosymbionts of insects like *Buchnera *and *Wolbachia *lack QS-related genes [24,25]. However, some secondary symbionts descending from pathogens like *Hamiltonella defensa *or *Sodalis glossinidius *do encode genes for AHL synthases [76]. Intracellular bacteria might have reduced needs for efficiency sensing, since secreted effectors are directly released into the cytosol [77]. In case of endofungal bacteria, classical QS signals might have become obsolete, because they might be quenched by the host [78]. However, we cannot rule out the possibility that *B. rhizoxinica *does harbor an exceptional type of QS system, since growth rate and formation of biofilm-like structures in pure culture seem to depend on cell density. Given the high number of NRPS gene clusters in the genome, it is conceivable that short cyclic peptides function as signal molecules as it was reported for *P. aeruginosa *[79].

## Conclusions

Many bacterial species colonize niches within eukaryotic host organisms as endosymbionts. Excellent studies of bacterial mutualists both of plants [51] and animals [80] have been described on genomic level in detail. Recent publications of symbiont genomes include symbionts of host species ranging from plants [81] to marine microalgae [82] and amoebae [83].

**Figure 4:**
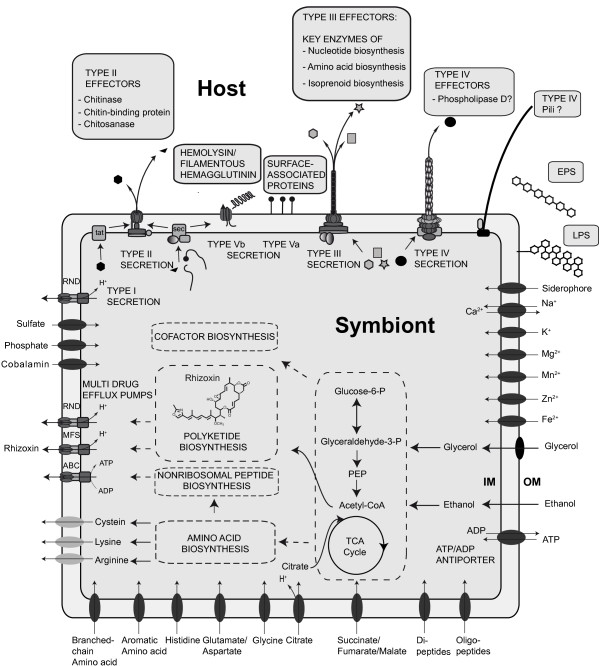
**Model of metabolic processes and host manipulating mechanisms deduced from the genome of *B. rhizoxinica***. Involvement of type III secretion system and LPS in symbiotic lifesytle have been demonstrated experimentally. Putative type III effectors were predicted by *in silico *methods. ABC = ATP-binding cassette superfamily, MFS = major facilitator superfamily, RND = resistance-nodulation-cell-division superfamily, EPS = exopolysaccharide, LPS = lipopolysaccharide, IM = inner membrane, OM = outer membrane.

However, symbionts of fungal species have long been a widely neglected area of research, although they have important implications for agriculture, biotechnology and food safety [13,84-86]. Here, we analyse the whole genome of *Burkholderia rhizoxinica*, which has been sequenced recently [23]. It is the first reported genome sequence of a mutualistic *Burkholderia *sp., and the first one of a bacterial endosymbiont of a fungus. The complete genome sequence provides unprecedented insights into the molecular equipment of a specialized symbiont that has most likely evolved via a parasitism-mutualism shift. *B. rhizoxinica *exhibits a 'genome in transition' [80] that is in an early stage of adaptation to the intracellular niche. Loss of sugar importers, in combination with the presence of citrate and dicarboxylate importers as well as a putative ATP/ADP antiporter suggest a metabolic adaptation towards an intracellular lifestyle (Figure 4). In contrast, *B. rhizoxincia *has retained biosynthetic pathways for the production of all proteinogenic amino acids and several important cofactors, which might be delivered to the fungus. Apart from the well-investigated rhizoxin biosynthesis gene cluster, *B. rhizoxinica *has a considerable biosynthetic potential for secondary metabolite production, namely NRPS gene clusters. Future projects may reveal the exact nature and bioactivity of these corresponding peptides. We speculate that some of these molecules play a role in communication processes, either among endobacteria themselves or between bacteria and fungi.

The fact that *B. rhizoxinica *survives within the host's cytosol and is able to invade the host under laboratory conditions suggested that *B. rhizoxinica *harbors specialized virulence-related factors for host manipulation and evasion of defense mechanisms. Indeed, we could identify members of the three multi-component secretion systems (type II, III and IV) and further putative factors for host manipulation and defense (Figure 4). Taken together, with this work, we hope to inspire two historically discrete scientific communities. On the one hand *B. rhizoxinica *can serve as a model system for microbial communication, infection and symbiosis. One the other hand, from a biotechnological point of view, the organism can be used for genome mining approaches and pathway engineering towards medically relevant rhizoxin derivatives. Overall, the whole genome sequence of *B. rhizoxinica *grants fascinating insights into the evolution and the genetic potential of endofungal bacteria and delivers an excellent basis for further investigations of bacterial-fungal interactions.

## Methods

### Strains and Culture Conditions

*Burkholderia rhizoxinica *B1 (HKI-0454) [10] was used in this study. Pure cultures of *B. rhizoxinica *were grown on standard nutrient agar (NaI) or in Tryptic Soy Broth (TSB) supplemented with 10 g L^-1 ^glycerol at 30°C and 120 rpm.

### Sequencing and Annotation

The complete genome of *B. rhizoxinica *was sequenced by the Sanger technology using a combination of a 2.8 kb shotgun library and a 36 kb cosmid library to an eight-fold average sequence coverage [23]. The reads were assembled into contigs using ARACHNE 3 Whole Genome Assembler [87]. Plasmid mate-pair information was exploited to resolve repetitive regions. Gaps were closed by sequencing on cosmid templates or combinatorial PCR. Circular topology of replicons was confirmed by PCR. Regions of low sequence quality or coverage were resequenced using cosmid templates to a minimum of two-fold coverage. The obtained three circular contigs were submitted to the ERGO™ (INTEGRATED GENOMICS, Chicago, Illinois, USA) annotation platform for automated annotation and pathway prediction. Selected loci were reannotated manually. The annotated nucleotide sequence of the *B. rhizoxinica *genome has been deposited in the EMBL database under the following accession numbers: EMBL:FR687359 (*Burkholderia rhizoxinica *HKI-0454, complete genome), EMBL:FR687360 (*Burkholderia rhizoxinica *HKI-0454 plasmid pBRH01, complete sequence), EMBL:FR687361 (*Burkholderia rhizoxinica *HKI-0454 plasmid pBRH02, complete sequence).

### Bioinformatic analyses

For annotation, pathway predictions, computation of feature statistics and comparative genomics, we used built-in tools of the ERGO™ (INTEGRATED GENOMICS, Chicago, Illinois, USA) platform. Synteny plots were created by the MUMMER3 program [88]. Signal Peptides for gram-negative organisms were predicted by SIGNALP 3.0 server [89]. Type III effectors were predicted with EFFECTIVET3[64] software package using the 'standard set' classification module (combining animal and plant specific effectors) and restriction value of 0.9999 (selective). Phylogenetic trees were obtained with MEGA 3.1 [90] using the built-in neighbor-joining algorithm. For assignment of BLASTP hits to the NCBI taxonomy, the program MEGAN 4 was used [91]. Prediction of horizontally acquired DNA based on computation of nucleotide compositional biases was performed with the ALIEN HUNTER program [92] provided by the SANGER WELCOME TRUST INSTITUTE.

## Authors' contributions

GL carried out genome assembly, annotation and analyses and drafted the manuscript. NM participated in genome analyses and revised the paper. LP-M participated in genome sequencing. SB participated in genome analysis. CH designed research and revised the manuscript. All authors read and approved the final manuscript.

## Supplementary Material

Additional file 1 **Figure S1: Phylogenetic trees of deduced ParB amino acid sequences (neighbor-joining method)**. Click here for file

Additional file 2 **Table S1: Prediction of antibiotic efflux pumps encoded in the genome**. Click here for file

Additional file 3 **Table S2: Prediction of Sec-exported proteins encoded in the genome**. Click here for file

Additional file 4 **Table S3: Prediction of type III secreted proteins encoded in the genome**. Click here for file
