# Supplementary material for: Evolution of an endofungal Lifestyle: Deductions from the Burkholderia rhizoxinica Genome
Source: BMC Genomics. 2011 May 4;12:210. doi: 10.1186/1471-2164-12-210 (PMC3102044; doi:10.1186/1471-2164-12-210)
Supplement: Additional file 1 — Figure S1: Phylogenetic trees of deduced ParB amino acid sequences (neighbor-joining method). [file 1471-2164-12-210-S1.DOC]

# Additional File 1


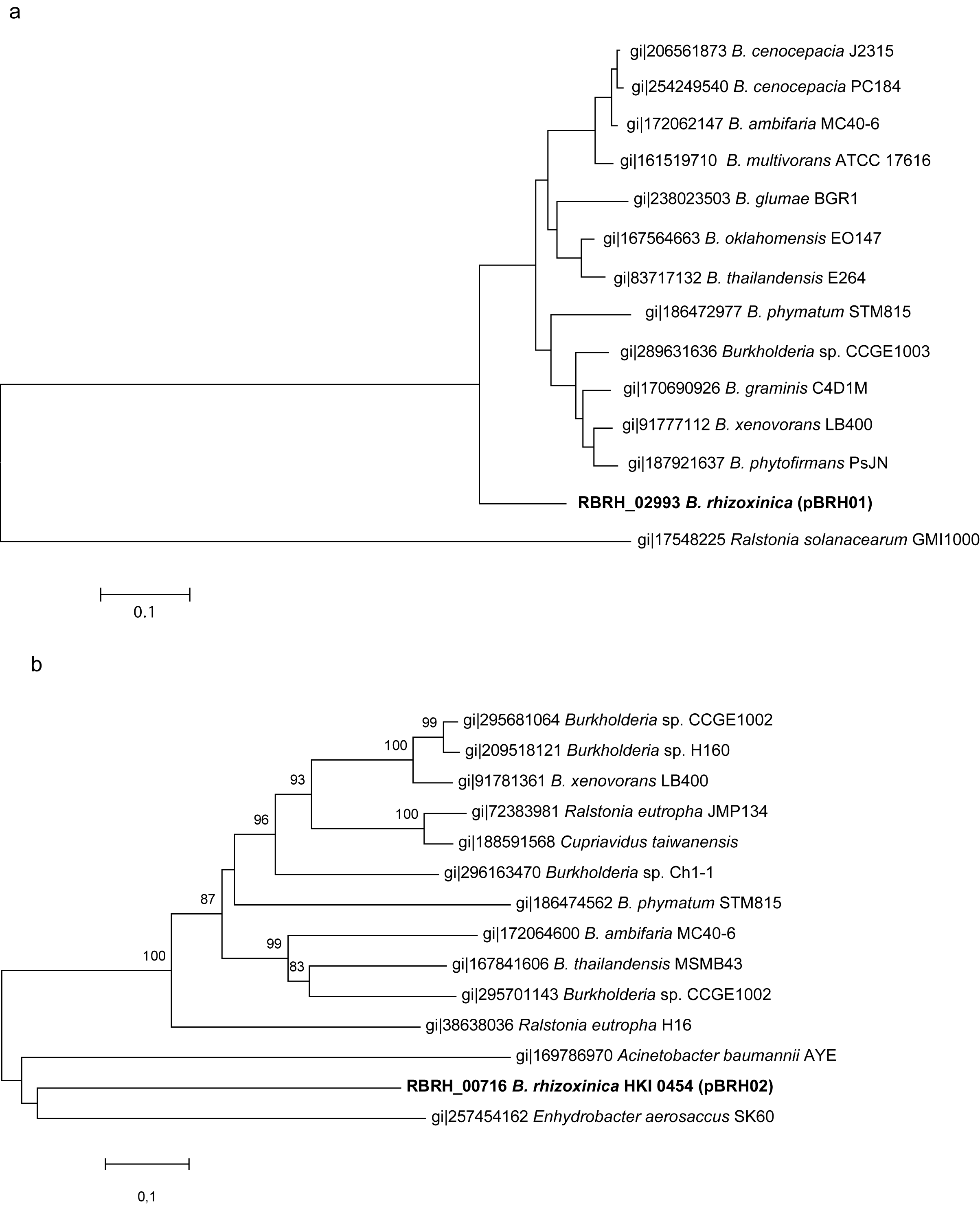


**Figure S1**

Phylogenetic trees of deduced ParB amino acid sequences (neighbor-joining method).

a: ParB (RBRH_02993) encoded on megaplasmid pBRH01. b: ParB (RBRH_00716) encoded on plasmid pBRH02.
