# Supplementary material for: Evolution of an endofungal Lifestyle: Deductions from the Burkholderia rhizoxinica Genome
Source: BMC Genomics. 2011 May 4;12:210. doi: 10.1186/1471-2164-12-210 (PMC3102044; doi:10.1186/1471-2164-12-210)
Supplement: Additional file 2 — Table S1: Prediction of antibiotic efflux pumps encoded in the genome. [file 1471-2164-12-210-S2.DOC]

# Additional File 2

**Table S1 Efflux Pumps.**

| **Number** | **ORFs** | **Annotation** |
| --- | --- | --- |
| **Resistance Nodulation Division (RND)** | | |
| 1 | RBRH_00529 RBRH_00528 RBRH_00527 | Acriflavin resistance periplasmic protein Acriflavin resistance plasma membrane protein Type I secretion outer membrane protein |
| 2 | RBRH_02982 RBRH_02980 | Acriflavin resistance periplasmic protein Acriflavin resistance plasma membrane protein |
| 3 | RBRH_01297 RBRH_01296 RBRH_01295 | Acriflavin resistance periplasmic protein Acriflavin resistance plasma membrane protein Type I secretion outer membrane protein |
| 4 | RBRH_03796 RBRH_03795 RBRH_03794 | Acriflavin resistance periplasmic protein Acriflavin resistance plasma membrane protein Acriflavin resistance plasma membrane protein |
| **ATP-Binding Cassette (ABC) Superfamily** | | |
| 1 | RBRH_00035 | Multidrug resistance ABC transporter ATP-binding and permease protein |
| 2 | RBRH_02398 | Multidrug resistance ABC transporter ATP-binding and permease protein |
| 3 | RBRH_02428 | Multidrug resistance ABC transporter ATP-binding and permease protein |
| 4 | RBRH_02553 | Multidrug resistance ABC transporter ATP-binding and permease protein |
| 5 | RBRH_03778 | Multidrug resistance ABC transporter ATP-binding and permease protein |
| 6 | RBRH_00040 RBRH_02901 RBRH_02902 | Macrolide-specific efflux protein macA Macrolide-specific ABC-type efflux carrier Type I secretion outer membrane protein |
| **Major Facilitator Superfamily (MFS)** | | |
| 1 | RBRH_00410 | Transporter, MFS superfamily |
| 2 | RBRH_01108 | Transporter, MFS superfamily |
| 3 | RBRH_01801 | Transporter, MFS superfamily |
| 4 | RBRH_02122 | Transporter, MFS superfamily |
| 5 | RBRH_00410 | Transporter, MFS superfamily |
