# Supplementary material for: Evolution of an endofungal Lifestyle: Deductions from the Burkholderia rhizoxinica Genome
Source: BMC Genomics. 2011 May 4;12:210. doi: 10.1186/1471-2164-12-210 (PMC3102044; doi:10.1186/1471-2164-12-210)
Supplement: Additional file 3 — Table S2: Prediction of Sec-exported proteins encoded in the genome. [file 1471-2164-12-210-S3.DOC]

# Additional File 3

**Table S2: Prediction of Sec-exported proteins**

| **Number** | **ORF** | **Annotation** |
| --- | --- | --- |
| 1 | RBRH_00025 | Sulfite-Dehydrogenase |
| 2 | RBRH_00782 | General secretion pathway protein |
| 3 | RBRH_00784 | Invasion protein |
| 4 | RBRH_00840 | Leucine-, isoleucine-, valine-, threonine-, and alanine-binding protein |
| 5 | RBRH_00360 |  |
| 6 | RBRH_01934 | Outer membrane usher protein CSSD |
| 7 | RBRH_03270 | Outer membrane porin protein 32 precursor |
| 8 | RBRH_03256 | UDP-N-acetylglucosamine--N-acetylmuramyl- (pentapeptide) pyrophosphoryl-undecaprenol N- acetylglucosamine transferase |
| 9 | RBRH_03214 | Outer membrane lipoprotein |
| 10 | RBRH_03205 | Thiol:disulfide interchange protein tlp |
| 11 | RBRH_01638 | 2-amino-4-hydroxy-6- hydroxymethyldihydropteridine pyrophosphokinase (EC 2.7.6.3) |
| 12 | RBRH_02856 | Chitosanase |
| 13 | RBRH_00949 |  |
| 14 | RBRH_00055 | Outer membrane protein |
| 15 | RBRH_01730 | ABC transporter substrate-binding protein |
| 16 | RBRH_01768 |  |
| 17 | RBRH_01769 |  |
| 18 | RBRH_04015 | Carbamoyl-phosphate synthase large chain (EC 6.3.5.5) |
| 19 | RBRH_01097 | Phosphate-binding protein |
| 20 | RBRH_02300 | Toluene transport system Ttg2D protein |
| 21 | RBRH_02274 | Cytochrome c1 |
| 22 | RBRH_02392 | Thiol:disulfide interchange protein DsbC |
| 23 | RBRH_02840 | Outer membrane porin protein 32 precursor |
| 24 | RBRH_02850 | Histidine-binding protein |
| 25 | RBRH_02876 | 34 kDa membrane antigen precursor |
| 26 | RBRH_02891 | NlpC/P60 family protein |
| 27 | RBRH_03705 | Hypothetical exported protein |
| 28 | RBRH_03655 |  |
| 29 | RBRH_03619 | Hypothetical protein |
| 30 | RBRH_03595 | Outer membrane porin protein 32 precursor |
| 31 | RBRH_03566 | Outer membrane protein Omp |
| 32 | RBRH_02656 | Leucine-, isoleucine-, valine-, threonine-, and alanine-binding protein |
| 33 | RBRH_01578 |  |
| 34 | RBRH_01248 | Hypothetical protein |
| 35 | RBRH_04147 |  |
| 36 | RBRH_03951 |  |
| 37 | RBRH_03961 | Tetratricopeptide repeat family protein |
| 38 | RBRH_03962 | Peptidyl-prolyl cis-trans isomerase (EC 5.2.1.8) |
| 39 | RBRH_03972 |  |
| 40 | RBRH_03795 | Acriflavin resistance plasma membrane protein |
| 41 | RBRH_01545 | Hypothetical exported protein |
| 42 | RBRH_01525 | Tol system periplasmic component YbgF |
| 43 | RBRH_00182 |  |
| 44 | RBRH_01134 |  |
| 45 | RBRH_01338 | Cytochrome c-554(548) |
| 46 | RBRH_02666 | Chitinase |
| 47 | RBRH_01417 |  |
| 48 | RBRH_01489 | Outer membrane siderophore receptor |
| 49 | RBRH_02079 | Glutamate/aspartate-binding protein |
| 50 | RBRH_02146 | Tetratricopeptide repeat family protein |
| 51 | RBRH_02160 | Anthranilate phosphoribosyltransferase (EC 2.4.2.18) |
| 52 | RBRH_02176 | ABC transporter substrate-binding protein |
| 53 | RBRH_02187 | Cytochrome c oxidase polypeptide II (EC 1.9.3.1) |
| 54 | RBRH_03076 | Transporter, LysE family |
| 55 | RBRH_02734 | SLA2 protein |
| 56 | RBRH_01265 | Hypothetical protein |
| 57 | RBRH_00974 |  |
| 58 | RBRH_04225 |  |
| 59 | RBRH_00301 | Arylesterase (EC 3.1.1.2) |
| 60 | RBRH_03435 | Toluene transport system Ttg2D protein |
| 61 | RBRH_02813 | Glycine betaine-binding protein |
| 62 | RBRH_02806 | Outer membrane porin protein 32 precursor |
| 63 | RBRH_04282 |  |
| 64 | RBRH_02976 |  |
| 65 | RBRH_03488 | Outer membrane porin protein 32 precursor |
| 66 | RBRH_03487 | Virulence protein |
| 67 | RBRH_00665 |  |
| 68 | RBRH_00661 |  |
| 69 | RBRH_00650 | Conjugal transfer protein TraN |
| 70 | RBRH_00648 | Conjugal transfer protein traF precursor |
| 71 | RBRH_00643 |  |
| 72 | RBRH_02902 | Type I secretion outer membrane protein |
| 73 | RBRH_04075 |  |
| 74 | RBRH_01547 | Chitin-binding protein |
| 75 | RBRH_00038 |  |
| 76 | RBRH_00007 | Hypothetical exported protein |
| 77 | RBRH_00797 | Two component system histidine kinase (EC 2.7.3.- ) |
| 78 | RBRH_00366 | Endonuclease (EC 3.1.-.-) |
| 79 | RBRH_01936 |  |
| 80 | RBRH_03263 | Cell division protein ftsL |
| 81 | RBRH_03253 | Cell division protein ftsQ |
| 82 | RBRH_04051 |  |
| 83 | RBRH_03203 | UDP-N-acetylmuramate:L-alanyl-gamma-D-glutamyl- meso-diaminopimelate ligase (EC 6.3.2.-) |
| 84 | RBRH_03325 | Hypothetical membrane spanning protein |
| 85 | RBRH_03318 | Virulence factor mviN |
| 86 | RBRH_03306 |  |
| 87 | RBRH_03137 | Cobalamin-binding protein |
| 88 | RBRH_01008 | GTP pyrophosphokinase (EC 2.7.6.5) / Guanosine- 3',5'-bis(Diphosphate) 3'-pyrophosphohydrolase (EC 3.1.7.2) |
| 89 | RBRH_02593 | Hypothetical membrane spanning protein |
| 90 | RBRH_02624 | Endopeptidase degP (EC 3.4.21.-) |
| 91 | RBRH_01110 | Membrane-bound lytic murein transglycosylase D precursor (EC 3.2.1.-) |
| 92 | RBRH_01104 |  |
| 93 | RBRH_01064 | Branched-chain amino acid transport system permease protein livH |
| 94 | RBRH_01157 | NAD(FAD)-utilizing dehydrogenases |
| 95 | RBRH_02341 | Periplasmic divalent cation tolerance protein CutA |
| 96 | RBRH_02340 | Thiol:disulfide interchange protein DsbD |
| 97 | RBRH_02318 | Outer membrane protein |
| 98 | RBRH_02301 | Lipoprotein |
| 99 | RBRH_02279 | Endopeptidase degP (EC 3.4.21.-) |
| 100 | RBRH_02944 | Transposase |
| 101 | RBRH_02947 | Fusaric acid resistance protein fusA |
| 102 | RBRH_02852 | Ketopantoate reductase PanE/ApbA family protein |
| 103 | RBRH_02877 | Hypothetical protein |
| 104 | RBRH_02880 | Hemin uptake protein hemP |
| 105 | RBRH_03701 |  |
| 106 | RBRH_03614 | Hypothetical protein |
| 107 | RBRH_02649 | Putative efflux protein |
| 108 | RBRH_01559 |  |
| 109 | RBRH_01579 | Ankyrin homolog precursor |
| 110 | RBRH_01604 | Peptidyl-prolyl cis-trans isomerase (EC 5.2.1.8) |
| 111 | RBRH_00102 | Lysine exporter protein |
| 112 | RBRH_00148 | Sulfate-binding protein |
| 113 | RBRH_04143 | NIFR3-like protein |
| 114 | RBRH_03837 | Regulatory protein |
| 115 | RBRH_03794 | Acriflavin resistance plasma membrane protein |
| 116 | RBRH_01528 | TolB protein |
| 117 | RBRH_02497 |  |
| 118 | RBRH_01337 | Cytochrome c-554(548) |
| 119 | RBRH_01478 |  |
| 120 | RBRH_01503 | Multidrug resistance protein |
| 121 | RBRH_01300 | Hypothetical protein |
| 122 | RBRH_01990 | Cytochrome c4 |
| 123 | RBRH_02017 |  |
| 124 | RBRH_02068 | Hypothetical protein |
| 125 | RBRH_02149 | Ribose-phosphate pyrophosphokinase (EC 2.7.6.1) |
| 126 | RBRH_02182 |  |
| 127 | RBRH_03116 | Cell division protein ftsN |
| 128 | RBRH_03115 | Thiol:disulfide interchange protein dsbA |
| 129 | RBRH_01279 |  |
| 130 | RBRH_00984 | Putative periplasmic protein |
| 131 | RBRH_00982 | Hypothetical exported protein |
| 132 | RBRH_03452 | Non-ribosomal peptide synthetase modules (EC 6.3.2.-) |
| 133 | RBRH_00467 |  |
| 134 | RBRH_00475 |  |
| 135 | RBRH_00509 |  |
| 136 | RBRH_00544 |  |
| 137 | RBRH_00308 |  |
| 138 | RBRH_00283 | Endopeptidase degP (EC 3.4.21.-) |
| 139 | RBRH_01821 | Embryo-specific protein |
| 140 | RBRH_02803 | Potassium-transporting ATPase A chain (EC 3.6.3.12) |
| 141 | RBRH_03541 | TypeIII secretion outer membrane protein SctC |
| 142 | RBRH_02987 | Ferric uptake regulation protein |
| 143 | RBRH_00664 |  |
| 144 | RBRH_00659 | Thiol:disulfide interchange protein DsbC |
| 145 | RBRH_00656 |  |
| 146 | RBRH_00654 | TraW protein |
| 147 | RBRH_00653 | TraU protein |
| 148 | RBRH_00649 |  |
| 149 | RBRH_00646 | TraH protein precursor |
| 150 | RBRH_00727 | Transposase |
