# Supplementary material for: Evolution of an endofungal Lifestyle: Deductions from the Burkholderia rhizoxinica Genome
Source: BMC Genomics. 2011 May 4;12:210. doi: 10.1186/1471-2164-12-210 (PMC3102044; doi:10.1186/1471-2164-12-210)
Supplement: Additional file 4 — Table S3: Prediction of type III secreted proteins encoded in the genome. [file 1471-2164-12-210-S4.DOC]

# Additional File 4

**Table S3: Prediction of type III secreted proteins**

| **Number** | **ORF** | **Annotation** |
| --- | --- | --- |
| 1 | RBRH_04056 |  |
| 2 | RBRH_04143 | NIFR3-like protein |
| 3 | RBRH_03397 | Acetylornithine deacetylase (EC 3.5.1.16) |
| 4 | RBRH_03792 |  |
| 5 | RBRH_00578 | Non-ribosomal peptide synthetase modules (EC 6.3.2.-) |
| 6 | RBRH_00204 | Adenosine deaminase (EC 3.5.4.4) |
| 7 | RBRH_02890 | Oligopeptide transport ATP-binding protein OppD/ Oligopeptide transport ATP-binding protein OppF |
| 8 | RBRH_03664 | Glucose-6-phosphate 1-dehydrogenase (EC 1.1.1.49) |
| 9 | RBRH_00169 | Transcriptional regulator, GntR family |
| 10 | RBRH_02389 | Uracil-DNA glycosylase (EC 3.2.2.-) |
| 11 | RBRH_03711 | Poly(3-hydroxyalkanoate) polymerase (EC 2.3.1.- ) |
| 12 | RBRH_02946 | Fusaric acid resistance protein fusB / Fusaric acid resistance protein fusC / Fusaric acid resistance protein fusD |
| 13 | RBRH_03580 | [protein-PII] uridylyltransferase (EC 2.7.7.59) |
| 14 | RBRH_03933 | TonB protein |
| 15 | RBRH_02420 | Transcriptional regulator, TetR family |
| 16 | RBRH_00422 | Lysine 2,3-aminomutase (EC 5.4.3.2) |
| 17 | RBRH_01270 | DNA processing protein |
| 18 | RBRH_03966 | RRNA methylase, SpoU family |
| 19 | RBRH_00781 | General secretion pathway protein E |
| 20 | RBRH_01304 |  |
| 21 | RBRH_03070 | Hypothetical cytosolic protein |
| 22 | RBRH_01302 | Transcriptional regulatory protein qseB |
| 23 | RBRH_00429 | Non-ribosomal peptide synthetase modules (EC 6.3.2.-) |
| 24 | RBRH_03577 |  |
| 25 | RBRH_03721 | Adenylosuccinate synthetase (EC 6.3.4.4) |
| 26 | RBRH_02310 | ABC transporter permease protein / ABC transporter ATP-binding protein |
| 27 | RBRH_03541 | Type IIIsecretion outer membrane protein SctC |
| 28 | RBRH_04279 | Non-ribosomal peptide synthetase modules (EC 6.3.2.-) |
| 29 | RBRH_00874 | Tail protein I |
| 30 | RBRH_01659 | (S)-2-Hydroxy-acid oxidase subunit GlcE (EC 1.1.3.15) |
| 31 | RBRH_04115 |  |
| 32 | RBRH_00655 |  |
| 33 | RBRH_01927 | Hypothetical protein |
| 34 | RBRH_03677 | Glucosyltransferase (EC 2.4.-.-) |
| 35 | RBRH_01757 | Fumarate hydratase (EC 4.2.1.2) |
| 36 | RBRH_00195 |  |
| 37 | RBRH_03189 | surface protein |
| 38 | RBRH_02101 | Hypothetical protein |
| 39 | RBRH_02943 | Hydroxypyruvate isomerase (EC 5.3.1.22) |
| 40 | RBRH_04269 |  |
| 41 | RBRH_01690 | Transposase |
| 42 | RBRH_03102 | Acylamino-acid-releasing enzyme (EC 3.4.19.1) |
| 43 | RBRH_01371 | Chromate transport protein |
| 44 | RBRH_01345 | DNA-3-methyladenine glycosylase (EC 3.2.2.20) |
| 45 | RBRH_03386 | Hypothetical protein |
| 46 | RBRH_00120 | Tail sheath protein gp18 |
| 47 | RBRH_01566 |  |
| 48 | RBRH_00033 |  |
| 49 | RBRH_00212 |  |
| 50 | RBRH_01786 | DNA polymerase III alpha subunit (EC 2.7.7.7) |
| 51 | RBRH_01921 |  |
| 52 | RBRH_04263 | Tetracycline resistance protein |
| 53 | RBRH_00400 |  |
| 54 | RBRH_01633 | tRNA delta(2)-Isopentenylpyrophosphate transferase (EC 2.5.1.8) |
| 55 | RBRH_00577 |  |
| 56 | RBRH_00274 | Non-ribosomal peptide synthetase modules (EC 6.3.2.-) |
| 57 | RBRH_02955 | Chorismate synthase (EC 4.2.3.5) |
| 58 | RBRH_01548 |  |
| 59 | RBRH_04093 |  |
| 60 | RBRH_00447 | Transposase |
| 61 | RBRH_03071 | Short chain dehydrogenase |
| 62 | RBRH_01759 | Na+ driven multidrug efflux pump |
| 63 | RBRH_01855 |  |
| 64 | RBRH_00297 |  |
| 65 | RBRH_03374 | Hypothetical protein |
| 66 | RBRH_03335 | Integrase/recombinase (XerD/RipX family) |
| 67 | RBRH_00598 | Insecticidal toxin complex protein TcaC |
| 68 | RBRH_01537 |  |
| 69 | RBRH_03467 |  |
| 70 | RBRH_02549 | Hypothetical protein |
| 71 | RBRH_00288 |  |
| 72 | RBRH_04231 |  |
| 73 | RBRH_03627 | Transcriptional regulator, MarR family |
| 74 | RBRH_02270 |  |
| 75 | RBRH_02804 | Potassium-transporting ATPase B chain (EC 3.6.3.12) |
| 76 | RBRH_00700 | Transposase |
| 77 | RBRH_02853 | Class II Aldolase and Adducin proteins |
| 78 | RBRH_00230 |  |
| 79 | RBRH_02564 | PIN domain |
| 80 | RBRH_03968 |  |
| 81 | RBRH_01207 |  |
| 82 | RBRH_01311 | Pyruvate kinase (EC 2.7.1.40) |
| 83 | RBRH_01611 | ABC transporter ATP-binding protein |
| 84 | RBRH_01040 | Hypothetical protein with endo/excinuclease domain |
| 85 | RBRH_02795 |  |
| 86 | RBRH_04129 |  |
| 87 | RBRH_00533 |  |
| 88 | RBRH_02080 | Glutamate dehydrogenase (EC 1.4.1.3) |
| 89 | RBRH_00057 | Phosphoglycolate phosphatase (EC 3.1.3.18) |
| 90 | RBRH_03485 |  |
| 91 | RBRH_00799 | Hypothetical protein |
| 92 | RBRH_03393 |  |
| 93 | RBRH_02109 | Queuosine biosynthesis protein QueF |
| 94 | RBRH_02237 |  |
| 95 | RBRH_02563 | Aspartate aminotransferase (EC 2.6.1.1) |
| 96 | RBRH_00892 | Tail protein X |
| 97 | RBRH_03867 | Non-ribosomal peptide synthetase modules (EC 6.3.2.-) |
| 98 | RBRH_02206 | Ammonium transporter / Methylammonium transporter |
| 99 | RBRH_01509 |  |
| 100 | RBRH_02508 | Acetolactate synthase large subunit (EC 2.2.1.6) |
| 101 | RBRH_02973 |  |
| 102 | RBRH_03609 | Oxygen-independent coproporphyrinogen-III oxidase (EC 1.3.99.22) |
| 103 | RBRH_03494 | Transporter |
| 104 | RBRH_01148 |  |
| 105 | RBRH_04020 |  |
| 106 | RBRH_00922 | DNA repair protein radC |
| 107 | RBRH_01869 | Hypothetical protein |
| 108 | RBRH_00365 | Ferredoxin--NADP reductase (EC 1.18.1.2) |
| 109 | RBRH_01099 | Dihydropteroate synthase (EC 2.5.1.15) |
| 110 | RBRH_04014 |  |
| 111 | RBRH_01796 | DNA topoisomerase I (EC 5.99.1.2) |
| 112 | RBRH_00235 | Hypothetical cytosolic protein |
| 113 | RBRH_00996 | AmpG protein |
| 114 | RBRH_02732 | Dipeptide transport ATP-binding protein dppD |
| 115 | RBRH_00726 | Transposase |
| 116 | RBRH_02631 |  |
| 117 | RBRH_03459 |  |
| 118 | RBRH_04203 |  |
| 119 | RBRH_01086 | FMN reductase (EC 1.5.1.29) |
| 120 | RBRH_01404 |  |
| 121 | RBRH_03563 |  |
| 122 | RBRH_02486 | Coproporphyrinogen III oxidase (EC 1.3.3.3) |
| 123 | RBRH_02976 |  |
| 124 | RBRH_02553 | Multidrug resistance ABC transporter ATP-binding and permease protein |
| 125 | RBRH_03963 | Peptidyl-prolyl cis-trans isomerase (EC 5.2.1.8) |
| 126 | RBRH_03197 | Phosphatidylglycerophosphatase A (EC 3.1.3.27) |
| 127 | RBRH_04296 |  |
| 128 | RBRH_01477 | Aspartate carbamoyltransferase (EC 2.1.3.2) |
| 129 | RBRH_01968 |  |
| 130 | RBRH_00009 | Methyltransferase (EC 2.1.1.-) |
| 131 | RBRH_02608 | Signal peptide peptidase sppA (EC 3.4.21.-) |
| 132 | RBRH_02482 | Septum formation protein Maf |
| 133 | RBRH_02692 | Benzaldehyde dehydrogenase [NAD+] (EC 1.2.1.28) |
| 134 | RBRH_01501 |  |
| 135 | RBRH_02470 | Phospholipid-lipopolysaccharide ABC transporter |
| 136 | RBRH_00991 | Acetylglutamate kinase (EC 2.7.2.8) |
| 137 | RBRH_00660 | Conjugal transfer protein traB |
| 138 | RBRH_00493 | Hydrolase-related protein |
| 139 | RBRH_01493 | Hypothetical protein |
| 140 | RBRH_03742 | Peptidoglycan-specific endopeptidase, M23 family |
| 141 | RBRH_00764 | Na+ driven multidrug efflux pump |
| 142 | RBRH_02386 | Anthranilate phosphoribosyltransferase (EC 2.4.2.18) |
| 143 | RBRH_03205 | Thiol:disulfide interchange protein tlpA |
| 144 | RBRH_03971 | DNA mismatch repair protein mutS |
| 145 | RBRH_03918 | Deoxyribodipyrimidine photolyase (EC 4.1.99.3) |
| 146 | RBRH_03395 | Hypothetical protein |
| 147 | RBRH_03732 | 1-hydroxy-2-methyl-2-(E)-butenyl 4-diphosphate synthase |
| 148 | RBRH_00719 | Reverse transcriptase (EC 2.7.7.49) |
| 149 | RBRH_02078 | Glutamate/aspartate transport system permease protein gltJ |
| 150 | RBRH_01946 | Lipopolysaccharide N- acetylglucosaminyltransferase |
| 151 | RBRH_03441 | Squalene--hopene cyclase (EC 5.4.99.17) |
| 152 | RBRH_01693 | Transposase |
| 153 | RBRH_02253 | Transposase |
| 154 | RBRH_03648 |  |
| 155 | RBRH_01761 |  |
| 156 | RBRH_02670 | Small heat shock protein |
| 157 | RBRH_01781 |  |
| 158 | RBRH_03430 | 1-Deoxy-D-xylulose 5-phosphate synthase (EC 2.2.1.7) |
| 159 | RBRH_02667 | 2-Nitropropane dioxygenase (EC 1.13.11.32) |
| 160 | RBRH_03717 | Myosin heavy chain |
| 161 | RBRH_02160 | Anthranilate phosphoribosyltransferase (EC 2.4.2.18) |
| 162 | RBRH_02666 | Chitinase (EC 3.2.1.14) |
| 163 | RBRH_03396 | Threonine dehydratase (EC 4.3.1.19) |
| 164 | RBRH_01218 |  |
| 165 | RBRH_01305 | Esterase (EC 3.1.1.1) |
| 166 | RBRH_04257 |  |
| 167 | RBRH_00325 | DNA repair protein RadA |
| 168 | RBRH_03433 |  |
| 169 | RBRH_01964 | 3-Oxoacyl-[acyl-carrier protein] reductase (EC 1.1.1.100) |
| 170 | RBRH_00226 | Serine (threonine) dehydratase (lantibiotic biosynthesis) / Lanthionine synthetase (lantibiotic biosynthesis) |
| 171 | RBRH_03730 | Hypothetical protein |
| 172 | RBRH_01746 | GTPase (EC 3.6.1.-) |
| 173 | RBRH_01949 | Nodulation protein nolNO (EC 2.1.3.-) |
| 174 | RBRH_00266 | Transposase |
| 175 | RBRH_02731 | Dipeptide transport system permease protein dppC |
| 176 | RBRH_01776 |  |
| 177 | RBRH_02938 |  |
| 178 | RBRH_00064 |  |
| 179 | RBRH_00534 |  |
| 180 | RBRH_02149 | Ribose-phosphate pyrophosphokinase (EC 2.7.6.1) |
| 181 | RBRH_02044 | Tail sheath protein Gp18 |
| 182 | RBRH_00100 | Hemagglutinin-like protein |
| 183 | RBRH_01670 | Paraquat-inducible protein B |
| 184 | RBRH_02518 | SSU ribosomal protein S15P |
| 185 | RBRH_02768 | Transcriptional regulator, TetR family |
| 186 | RBRH_03217 | Ribonucleoside-diphosphate reductase beta chain (EC 1.17.4.1) |
| 187 | RBRH_01601 | Peptide methionine sulfoxide reductase msrB (EC 1.8.4.11) |
| 188 | RBRH_01941 |  |
| 189 | RBRH_01068 |  |
| 190 | RBRH_01308 | Phosphoribosylaminoimidazole carboxylase carboxyltransferase subunit (EC 4.1.1.21) |
| 191 | RBRH_00542 | Bacitracin transport permease protein bcrC homolog |
| 192 | RBRH_03316 | Hemolysin activator protein precursor |
| 193 | RBRH_02186 | Cytochrome c oxidase polypeptide I (EC 1.9.3.1) |
| 194 | RBRH_01953 |  |
| 195 | RBRH_03110 | Zinc metallohydrolase, glyoxalase II family |
| 196 | RBRH_00607 |  |
| 197 | RBRH_02037 |  |
| 198 | RBRH_01517 | Ribosomal-protein-serine acetyltransferase (EC 2.3.1.-) |
| 199 | RBRH_01534 | Serine hydroxymethyltransferase (EC 2.1.2.1) |
| 200 | RBRH_01913 | Transposase |
